# Supplementary material for: Communication of an Abnormal Metabolic New-Born Screening Result in The Netherlands: The Parental Perspective
Source: Nutrients. 2022 Sep 23;14(19):3961. doi: 10.3390/nu14193961 (PMC9571573; doi:10.3390/nu14193961)
Supplement: Supplementary file 1 [file nutrients-14-03961-s001.zip › nutrients-1895361-supplementary.pdf]

Questionnaire Parental Satisfaction Newborn Screening (NBS)

(translated from Dutch to English)

1. Do you have experience with a general practitioner (GP) who communicated the abnormal NBS result of PKU to you and/or your partner?
2. In what year did this take place?
3. Are you satisfied with the way the GP communicated the abnormal NBS result to you and/or your partner?
4. What makes you satisfied with the communication of the GP?
5. What makes you unsatisfied with the communication of the GP?
6. Who contacted you?
    a. Own GP
    b. Replacing GP
    c. GP's assistant
    d. Other
7. How was the NBS result communicated?
    a. Via telephone
    b. With a consult
    c. GP at home
    d. Other
8. Did the GP had contact with the metabolic paediatrician during the conversation?
9. Did the GP had contact with the metabolic paediatrician before the conversation?
10. Did you discuss the communication of the abnormal NBS result with your GP afterwards?
11. What did the GP tell you?
12. Did you receive clear instructions from the GP on where to go to; e.g. which hospital, which department and at what time?
13. Was de reception in the hospital good?
14. Do you want to share your experience with the NBS result?
15. Do you have any ideas on how the communication of an abnormal NBS result could be improved?
